# Trust, Governance, and the Covid‐19 Pandemic: an Explainer using Longitudinal Data from the United Kingdom

**DOI:** 10.1111/1467-923X.13131

**Published:** 2022-04-16

**Authors:** James Weinberg

**Keywords:** political trust, Covid‐19, policy performance, government satisfaction, politicians, political behaviour

## Abstract

Crises like the Covid‐19 pandemic place an added premium on the social contract underpinning principal‐agent relations in representative democracies, which relies, at a fundamental level, on conditional trust judgements *by* those without power *in* those with decision‐making authority to act in their better interests. Existing studies of political trust during the pandemic suggest that it has been both a symptom of government activity as well as a cause of its success or failure. Presenting original longitudinal data collected from UK citizens at the start of the pandemic and again twenty months later, the article teases apart these dynamics and their implications. It shows, for example, that the public became less trusting and more distrusting of politicians during this unique moment, and that these trends are strongly linked to performance evaluations of the UK government as well as public compliance with mandatory and non‐mandatory policies such as vaccination and mask wearing.

## Introduction

THE GLOBAL pandemic of novel coronavirus SARS‐CoV‐2, which causes the respiratory disease otherwise known as Covid‐19, placed an added premium on the social contract that underwrites governor‐governed relations and, in particular, the political trust of citizens in politicians with legislative authority. As Daniel Devine and his colleagues at the TrustGov project sagely noted in the early months of the crisis, trust ‘could be seen as essential to facilitating good governance of the pandemic’.^1^ Testifying to this prediction, existing research finds consistent positive correlations between individuals’ political trust and their support for government policies as well as actual behavioural change.^2^ A second tranche of research has focused more on the *causes* as opposed to *symptoms* of political trust during the pandemic. Here, a mixture of cross‐sectional studies and meta‐analyses of polling statistics suggest a boom‐and‐bust cycle of political trust after an early rally‐round‐the‐flag effect.^3^

Whilst informative, these studies only offer a snapshot of the relevance of political trust during the pandemic or, where they analyse changes over time, they draw on multiple sources of data that use different theories and measures of political trust that are not directly comparable. Advancing this existing pool of research, I offer an illustrated explainer of the links between trust and Covid‐19 governance using a longitudinal survey conducted across twenty months of the pandemic with a nationally representative sample of UK citizens.^4^ The added benefits of longitudinal data are two‐fold. On one hand, they allow an effective examination of changes in political trust over time by sampling the same individuals, using the same measures, at multiple intervals across the crisis. On the other hand, they provide much greater accuracy and power when it comes to drawing conclusions about (a) how exactly people's levels of political trust might have been impacted by the pandemic (and the governance thereof), and (b) how such changes might have impeded or facilitated subsequent policy success by influencing people's behaviour.

To address these questions, participants were surveyed on 2–4 April 2020 and again on 10–13 December 2021. Between these two dates, the UK's approach to managing the pandemic changed drastically. Prior to wave one of data collection, the government had pursued a strategy of behavioural ‘nudging’ as a calculated move to encourage ‘herd immunity’. By early March 2020, this singled the UK out as a control case of sorts at a time when other nations with large numbers of cases (China, South Korea, Italy and Iran) as well as those with relatively few (Ireland, Norway and Denmark) had implemented stricter lockdown measures. With the number of Covid‐19 cases in the UK rising rapidly, the number of deaths mounting, and the projected pressures on the NHS monumental, the government reversed its approach in favour of stringent protocols to keep people at home. The Coronavirus Act 2020, which received Royal Assent on 25 March, was rushed through Parliament in just four sitting days. The Act allowed for exceptional new forms of resourcing and funding for public bodies and local authorities as well as provisions for temporary and exceptional alterations to workers’ rights and the use of legal and policing powers to prohibit transmission of the virus.^5^ Therefore, at the time of sampling, participants were just one week into an economic and social ‘lockdown’ that was unprecedented in the postwar era.

Between waves one and two of the survey, the UK would yo‐yo between lifting and tightening restrictions on public liberties in response to new variants of the virus; a strategy that Prime Minister Boris Johnson incidentally described as a ‘whack‐a‐mole’ approach. By the time that participants were surveyed in December 2021, the UK had recorded the highest Covid‐related death toll in Europe and the fourth highest number of cases in the world. And in spite of an objectively successful vaccination programme, a new variant of Covid‐19 known as Omicron was also creating some concern about vaccine efficacy. Just two days before the survey fielded, the government reacted to this uncertainty by reintroducing a number of laws and guidance measures under the title ‘Plan B’. In what follows, I use data collected at these two time points to tease out the links between government performance during the pandemic, levels of political trust, and compliance with voluntary and mandatory policies on vaccination and mask wearing.

## Levels of trust, distrust and mistrust

Trust is a slippery term with as many definitions as there are sub‐disciplines in social science. It is worth being clear, therefore, about what exactly is meant when talking of trust in this article. Specifically, political trust is used here to denote an evaluative attitude grounded in cognition, affect and behavioural intentions. It is entirely relational (that is, it occurs between two people or a person and an institution); it is characterised by multi‐faceted judgements about the ‘trustworthiness’ of a specific trustee in a specific context of action; and it is interrelated to, but distinguishable from, its brethren concepts of *dis*trust and *mis*trust. Whilst trust is based on positive evaluations of a trustee's competence, benevolence and integrity, and thus facilitates vulnerability and cooperative behaviours, distrust is based on negative evaluations that invoke an expectation of harm or betrayal and thus elicit very different actions to manage risk. Mistrust, by contrast, is a more exacting form of trust based on heightened caution and increased scrutiny of a trustee. These concepts can be understood with reference to some of the items used to measure them in this study. For example:

- MPs are doing their best to represent other people's interests, including yours. (*TRUST*)
- MPs are happy to make promises at elections, but then forget them afterwards. (*DISTRUST)*
- You double‐check what MPs tell you in case of misleading information. (*MISTRUST*)

Participants were asked to agree or disagree with these judgements about MPs using a seven‐point scale (strongly disagree—strongly agree). In total, the survey contained twelve items measuring trust, nine items measuring distrust, and three items measuring mistrust. Participants answered identical items in both waves of data collection.^6^

On average, levels of political trust dropped by 13 per cent between wave one and wave two of this study (to a mean score of 3.08/7), whilst average levels of distrust increased by 7 per cent (to a mean score of 5.51/7). Average differences in mistrust were negligible and statistically insignificant (mean score of 4.21/7 in wave two). Put another way, the UK public became less positive, more cynical, and equally sceptical about their national politicians during this time of crisis.

As noted above, however, trust judgements are target specific. Whilst participants were asked to self‐report their attitudes towards ‘MPs’, it is well‐known that such items induce heuristic thinking. After responding to all twenty‐four items, participants were asked to self‐report the ‘target’ that had guided their answers (that is, *who* or *what* had they been thinking about when they read the words ‘Members of Parliament’?). Four dominant categories of trustee emerged across both waves of data collection: ‘all MPs’ (N = 263), ‘the UK government’ (N = 210), ‘my local MP’ (N = 63), and ‘the Prime Minister’ (N = 134).

**Figure 1:**
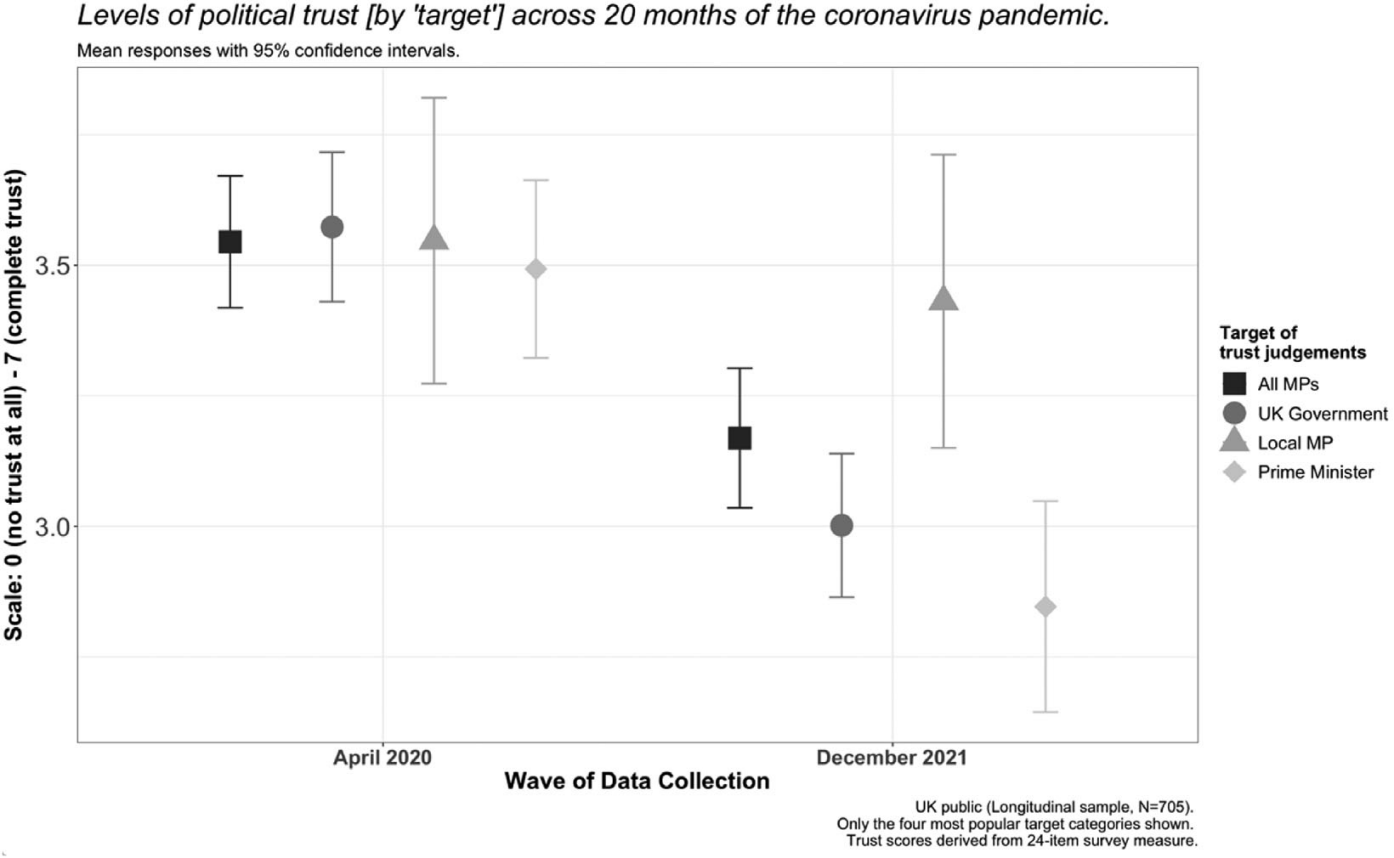
Political trust during the Covid‐19 pandemic

Reassessing levels of political trust according to these categories, it is possible to discern a more nuanced picture of its trajectory over time (Figure 1). Whilst there were no significant differences between participants’ responses to the survey items according to these categories in April 2020, there were distinct variations in the amount by which each changed over the following twenty months. To be precise, political trust in local MPs remained broadly comparable over time, but levels of trust in MPs as a collective body, in the UK government and in Prime Minister Boris Johnson, all dropped meaningfully. The largest decrease, 19 per cent, was recorded for trust judgements about the Prime Minister. These aggregate trends were also recorded among voters of all partisan affiliations. In fact, average levels of political trust in the UK government (as a self‐reported target of trust judgements) dropped equally by 16 per cent amongst Conservative voters and opposition voters.

## Explaining changes in political trust

Having established that levels of political trust and distrust changed during the Covid‐19 pandemic, the more interesting question is: why? Two plausible hypotheses are that (a) trust was artificially inflated at the start of the pandemic and then naturally returned to its pre‐pandemic malaise by the end of 2021, or (b) the initial upturn in political trust dissipated as a direct consequence of the political management of the crisis. A dominant trust‐as‐evaluation tradition in political science supports the latter supposition. The compelling conclusion of existing research is that, as Beate Huseby puts it, ‘poor performance in salient political issues leads to negative evaluations of government performance, which in turn influences citizens’ support for the political system’.^7^ The pandemic represents a rare natural experiment for testing the trust‐as‐evaluation tradition with the current dataset. Participants were surveyed just one week after the start of the UK's first national lockdown and then again forty‐eight hours after ‘Plan B’ had been announced in response to the Omicron variant during the fourth wave of the pandemic. Between the two waves of data collection, UK citizens were not only hyper‐aware of government policy on Covid‐19, and thus what was being asked of them, but also witnessed a number of symbolic political scandals linked directly to those policies (for example, breaches of lockdown restrictions by senior government officials and elected politicians).

**Figure 2:**
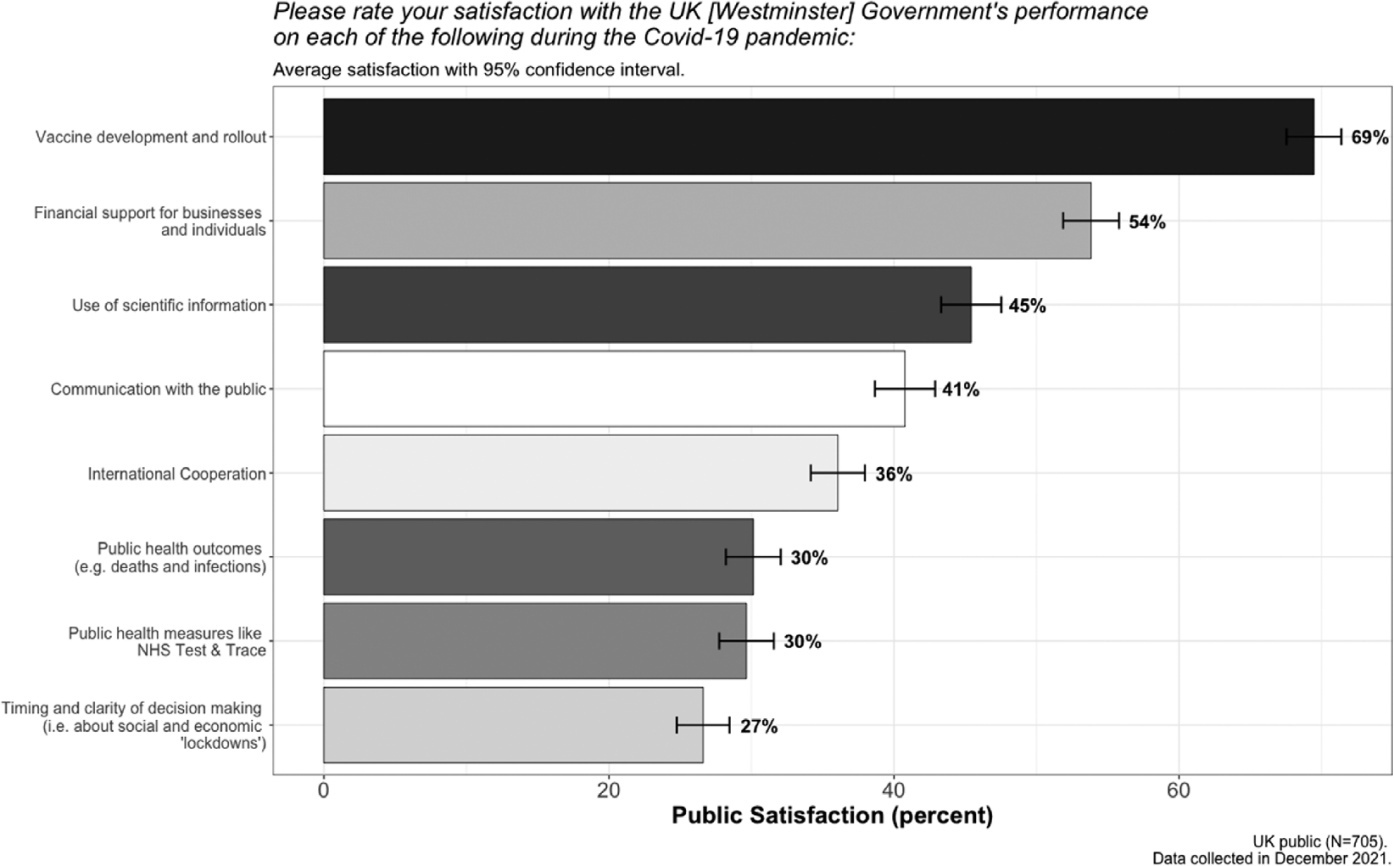
Public satisfaction with Covid‐19 governance in the UK

Figure 2 shows public satisfaction with the government's performance during the pandemic as measured across eight salient indicators. For six of these eight metrics, public satisfaction does not rise above 50 per cent. Whilst the UK public was relatively satisfied with the government's vaccination programme and its financial support schemes, people were particularly *un*satisfied with the timing and clarity of its decision making, public health measures such as NHS Test & Trace, and public health outcomes like the number of deaths and infections that were recorded. Whilst previous studies of this crisis have shown cross‐sectional correlations between government satisfaction and political trust, I now look at the relationship between these ratings and the *amount of change* in individual levels of political trust reported over the course of the pandemic. In doing so, it is possible to get a better idea of the causal relationship between these two variables.

**Figure 3:**
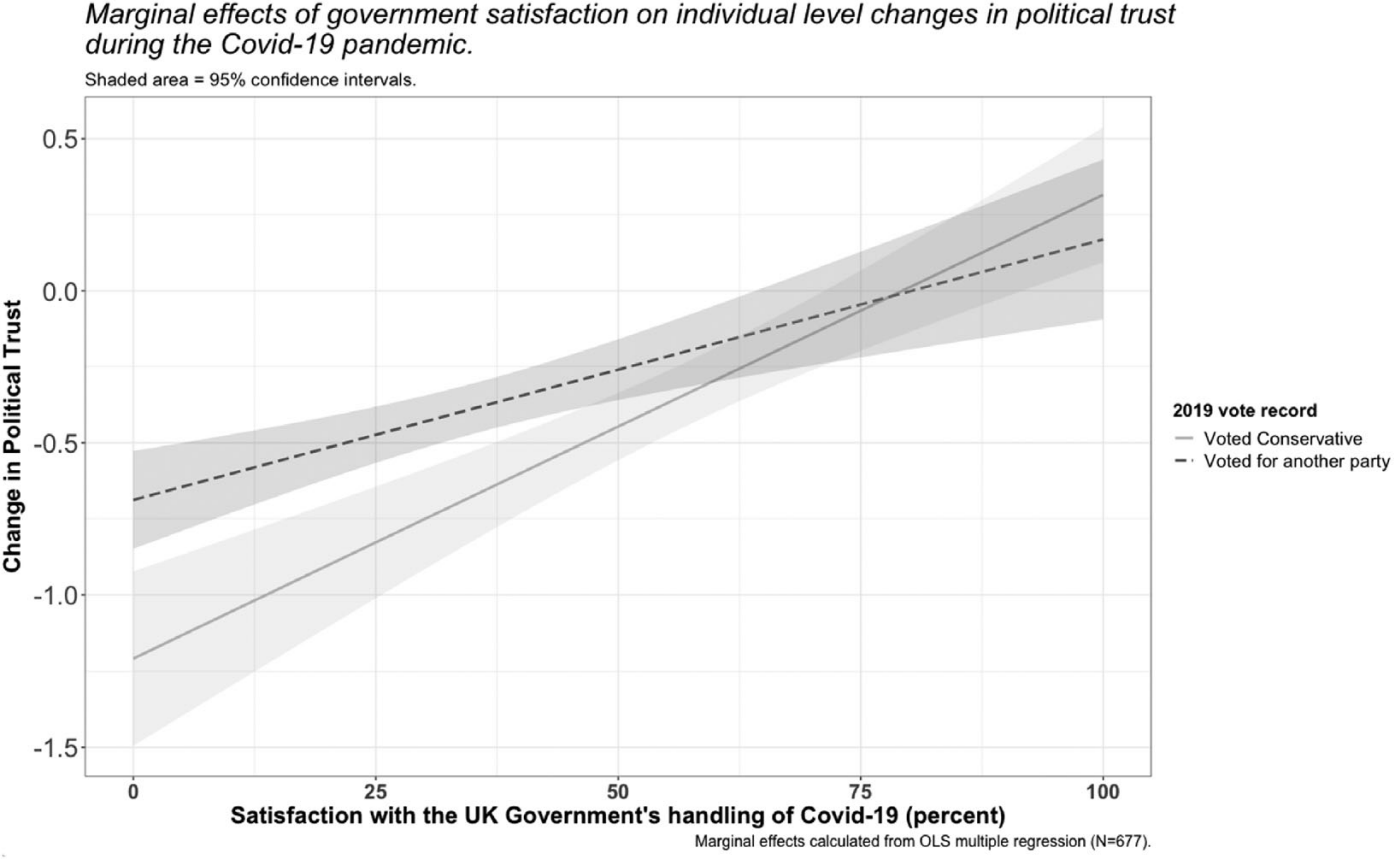
The impact of public satisfaction with the UK government's handling of the Covid‐19 pandemic on changes in political trust

I start by combining participants’ satisfaction ratings into a single aggregate score (mean = 41.8 per cent, standard deviation = 21.7). I then assess the impact of these satisfaction ratings on the change in participants’ political trust over time (see Figure 3). I control for participants’ various trust ‘targets’ as well as their partisanship (specifically whether or not they voted for the incumbent Conservative government in the UK's 2019 general election). In doing so, I account for any partisan bias that may arise from motivated reasoning. Motivated reasoning is a well‐researched theory originating in cognitive science and social psychology that describes the ways in which people (often subconsciously) employ biases and heuristics to avoid cognitive dissonance. In other words, they seek out or process information positively if it conforms to prior beliefs and process information negatively if it does not. In politics, motivated reasoning can also manifest through selective attributions of responsibility.^8^ For example, Conservative Party voters may be more likely than opposition voters to deflect blame away from the incumbent government for policy failures during the Covid‐19 pandemic and more likely to attribute credit to it for policy successes. This assumption is reflected in higher satisfaction ratings—of the government's policy performance on Covid—among its 2019 voters (mean = 54 per cent, N = 212) than among voters for other parties (mean = 36 per cent, N = 455). As such, the relationship between government satisfaction and changes in political trust and distrust becomes contingent on an added layer of psychological processing.

In line with the trust‐as‐evaluation thesis, I find a linear relationship between participants’ satisfaction ratings and the amount of change in their political trust (see Figure 3 for marginal effects).^9^ As satisfaction with the government's performance during the pandemic decreases, so too do people's evaluations of politicians’ trustworthiness. As anticipated, this relationship is partly contingent on partisanship. Yet, far from fulfilling the theoretical expectations of the literature on motivated reasoning, evaluations of the government's performance appear to have had an equal, if not slightly stronger, effect on the trust of Conservative voters compared to voters for other parties. Having made themselves vulnerable to the Conservative Party in 2019 by giving their vote (that is, an act of trust), it is plausible that these voters felt a greater sense of betrayal over *any* admission of policy failure by the government. These results also matter because they suggest that trust is labile in response to political decisions taken in a crisis scenario. In turn, the practical implication is that governments have agency to change public perceptions—contra rote claims that politicians are universally hated—and must think carefully about how they use that agency to manage these psychological resources responsibly.

## Trust as a resource in the pandemic

Whether or not the government's handling of the Covid‐19 pandemic impacted political trust is only important, to some extent, if political trust also impacts public behaviour in a way that impedes or facilitates vertical coordination (for example, policy compliance and responsiveness). The central finding from existing research is that people withdraw their support for policies—especially those entailing risk or sacrifice—when they do not trust those in power. Political trust is, then, a heuristic that aids people's prospective judgements about whether or not it will be safe and beneficial to support a policy.^10^ Or put another way, people seek warrants (that is, evidence of trustworthiness) by which to justify making themselves vulnerable to politicians in situations involving uncertainty. For this reason, political trust is even more critical during a crisis scenario when non‐compliance with government activity may well cost lives. Early cross‐sectional evidence from the Covid‐19 pandemic suggests that trust, and in some cases mistrust, was positively correlated with public compliance over social and economic lockdown measures (for example, stay‐at‐home orders, social distancing requirements, and travel bans).^11^

I re‐test these assumptions here by analysing the relationship between individual‐level changes in political trust and compliance with two key public health measures in the UK as of December 2021: vaccinations and mask wearing.^12^ Very early in the pandemic, it was widely acknowledged that vaccinations would be a necessary, if not necessarily sufficient, condition for successfully navigating through and out of the Covid‐19 crisis. The UK government invested heavily—both financially and rhetorically—in this assertion. By the end of December 2020, the Joint Committee on Vaccination (JCVI) had approved two vaccines for dissemination. Twelve months later, more than 51 million people in the UK had received their first vaccine, almost 47 million had received their second vaccine, and just under 23 million had received a booster vaccine.^13^ Reflective of these statistics, 90 per cent of participants surveyed for this study had been vaccinated at least once by December 2021.

**Figure 4:**
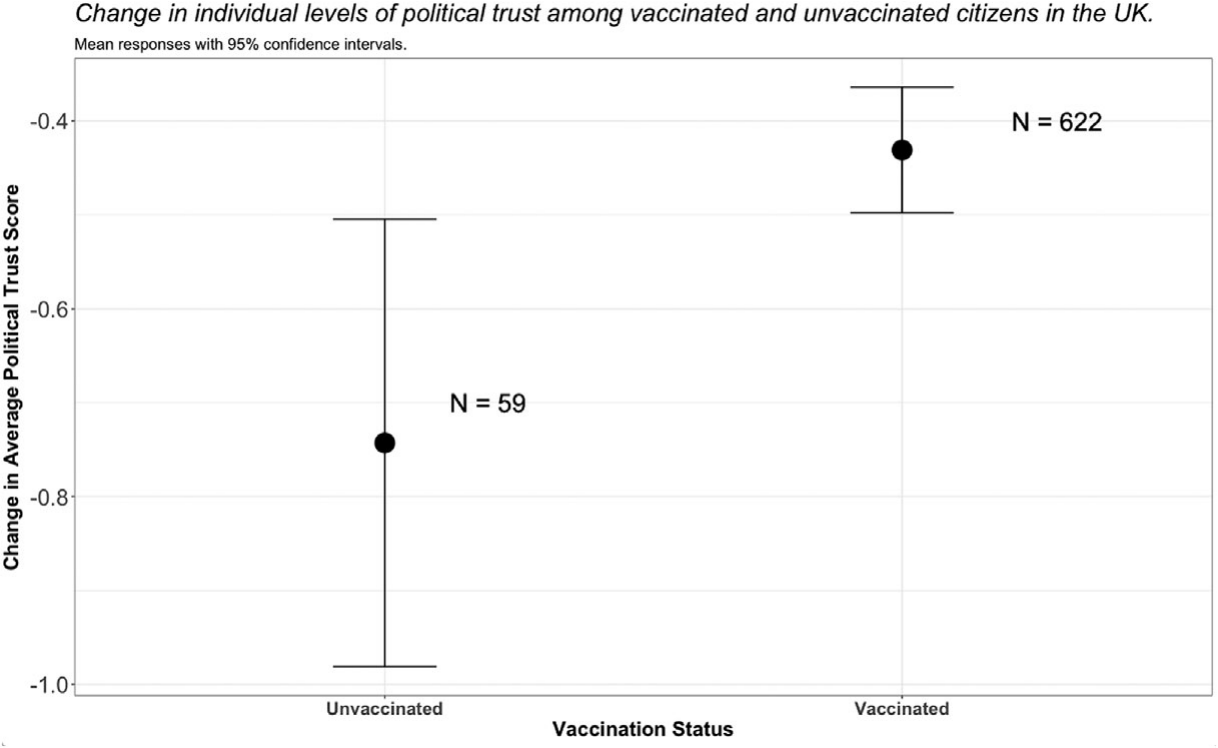
Political trust and vaccinations in the UK

So what about the remaining 10 per cent? Can political trust help to explain why, despite being offered the vaccine, some people did not accept it? Whilst the number of unvaccinated participants in this study is too small to offer robust inferential analysis, descriptive comparisons *do* suggest a relationship between these two variables (Figure 4). To be specific, unvaccinated members of the public had lost considerably more trust in UK politicians during the pandemic—almost twice as much on average—than those who were vaccinated. An inverted trend was found for political distrust, although the difference between the two groups was not as large. These patterns are theoretically intuitive. The more that citizens lost faith in the competence, benevolence and integrity of those passing legislation on Covid‐19 (that is, their trustworthiness), the less likely they were to accept the perceived risks of complying with politicians’ invocations to ‘get jabbed’.

**Figure 5:**
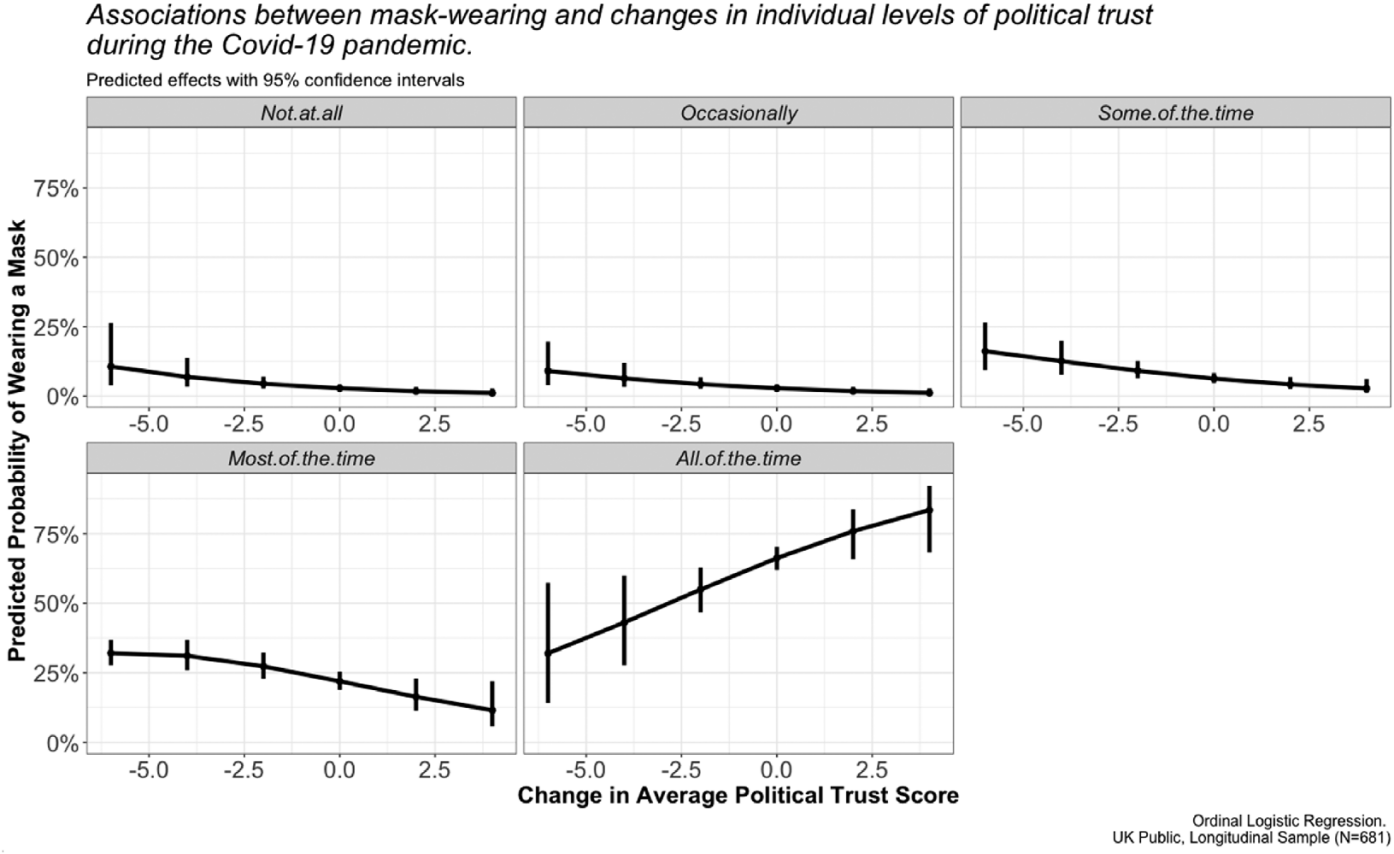
Political trust and mask wearing in the UK

Next, I explore the relationship between political trust and mask wearing. When the UK government announced ‘Plan B’ on 8 December 2021, it became mandatory to wear a face covering in most indoor public venues as well as on public transport. This decision was taken in response to rising concerns about case numbers of the new Omicron variant of Covid‐19, which were doubling every two to three days at the time of the announcement. Yet, just days later (at the time of sampling), not everybody was complying with this new legal obligation. As depicted in Figure 5, political trust may help to make sense of this decision. The more political trust that people had lost over the prior twenty months of the pandemic, the less likely they were to be wearing a mask ‘all’ of the time (where and when necessary). If elevated levels of trust did indeed lead to enhanced policy compliance at the start of the pandemic (as evidenced elsewhere), it seems that such support was not well utilised or rewarded by the UK government. Not only did most members of the public subsequently downgrade their perceptions of politicians’ trustworthiness (see previous sections), but these changes in levels of political trust impacted support for/opposition to key measures that were being taken by the government as the country navigated its way through the final stages of the pandemic's immediate threat.^14^

## Conclusions and implications

When it comes to understanding political leadership, trust and trustworthiness are critical barometers of success. In crisis scenarios especially, political trust is symbolic of people's faith in the capacity of leaders to look after their better interests and, in turn, it becomes a key asset that politicians can draw upon to mobilise a community, or even a nation, in the face of grave threats and collective challenges. In this brief explainer, I have attempted to illustrate these claims through an empirical case study of the UK's handling of the Covid‐19 pandemic. Using longitudinal data collected over twenty months, I present evidence of both the trust‐as‐evaluation and trust‐as‐resource theses. On one hand, the public was distinctly unsatisfied with the government's policy performance on Covid‐19, and this dissatisfaction was strongly related to decreased levels of trust in politicians generally. On the other hand, these changes in individual levels of political trust appear to have mattered for the success of policy announcements during the fourth wave of the pandemic. As levels of trust declined, so too did the chances that people were getting vaccinated and wearing face coverings in public spaces.^15^

It is worth noting that political trust declined more over the course of the pandemic than distrust increased and, in turn, levels of mistrust remained steady. For students of politics, and specifically trust in politics, this is an important finding that suggests these theoretical constructs, whist interlinked, may remain conceptually and empirically distinct. Accepting that trust is always relational and target specific, I also show the merit of digging deeper into existing survey measures of these concepts. Put simply, long‐running surveys—including national election studies in the UK and beyond—need to be mindful of the heuristics that respondents use when answering items about ‘politicians’, ‘MPs’, or institutional markers like ‘parliament’. There is a lot more to be read into our data when we tease out the trustees that people use to answer questions on political trust and, in turn, it is important that we control for these variations in subsequent analyses to account for related measurement error. In this instance, participants’ trust targets were instructive insofar as they helped to unpick trends in political trust over time. Specifically, the UK government and the Prime Minister, Boris Johnson, appear to have suffered the largest hit to political trust, and the largest boon to political distrust, during the pandemic. Dependent on within—as opposed to between—participant comparisons, these findings suggest that political leaders are most exposed to shifts in public opinion during crisis scenarios, whilst notions of a personal vote, whereby MPs can cultivate strong relationships in their constituencies, may well protect backbench MPs.

Theoretically, the findings presented here also raise compelling questions about the partisan dynamic of political trust. It is often accepted that partisan bias in performance evaluations of incumbent governments might go on to create a partisan bias in citizens’ judgements about the trustworthiness of said government and/or politicians more broadly. Focusing on the present case study, this supposition was partly ratified by participants’ satisfaction ratings of the Conservative Government's performance on Covid‐19, which were notably lower among citizens who did not vote for them in the 2019 general election. However, there was no discernible partisan difference in the strength of the association between these ratings and changes to participants’ political trust over the pandemic. This would suggest that even when government supporters and opposition voters do not evaluate government performance to an *equal degree*, negative evaluations may nevertheless go on to cause *as much or more* damage to the political trust of in‐partisans. Put another way, in‐partisans may react more strongly to admissions of poor performance when their party holds power, not least because such admissions are symbolic of a betrayal of the trust expressed in their prior vote. These findings may help to explain why the Conservative Party lost the North Shropshire by‐election—a safe seat that they had held for nearly 200 years—less than one week after the second wave of data for this study was collected.

In damaging the public's political trust, it is clear that politicians also damage the legitimacy of their office and the credibility of the policies that are designed to keep everyone safe in a crisis scenario. Where trust decreases, and distrust increases, people are more likely passively to disengage from or, even worse, actively dissociate themselves from, important policies such as mask wearing and vaccination. At the time of writing, the Metropolitan Police has just issued fines over revelations about social gatherings held inside 10 Downing Street and Whitehall, which were convened in contravention of coronavirus laws enforced elsewhere in the country. This so‐called ‘Partygate’ scandal is likely to diminish further citizens’ judgements about the benevolence and integrity of the country's leading politicians and, in turn, reinforce the trends in political trust and distrust reported in this article. In the medium term, it is plausible that this episode will produce the equivalent of a trust hangover insofar as the public's trust in politicians, and politics more broadly, remains in deficit to their distrust. Politically, an extended trust deficit may be particularly damaging for the Conservative Party. Practically, it may be damaging for levels of public cooperation and engagement through the post‐pandemic recovery period, which will be characterised by significant policy challenges in the domains of health, education and commerce (to name a few). Current and future administrations will need to invest heavily in rebuilding trust and thus overturning this deficit in order to govern effectively through these substantial difficulties as well as the next ‘unknown unknown’.

## Notes

^1^
D. Devine, J. Gaskell, W. Jennings, et al., ‘Trust and the coronavirus pandemic: what are the consequences of and for trust? An early review of the literature’, *Political Studies Review*, vol. 19, no. 2, 2020, pp. 274–285.

^2^
See, for example, W. Jennings, G. Stoker, V. Valgarsson, et al., ‘How trust, mistrust and distrust shape the governance of the COVID‐19 crisis’, *Journal of European Public Policy*, vol. 28, no. 8, 2021, pp. 1174–1196.

^3^
B. Davies, F. Lalot, L. Peitz, et al., ‘Changes in political trust in Britain during the COVID‐19 pandemic in 2020: integrated public opinion evidence and implications’, *Humanities & Social Sciences Communications*, vol. 8, no. 166, 2021; https://doi.org/10.1057/s41599-021-00850-6 (accessed 7 April 2022).

^4^
Participants were recruited via the crowd‐working platform Prolific Academic, which maintains survey panels in the US and UK, and this particular survey module was part of a larger study of trust and governance taking place between 2019 and 2022. A total of 1045 participants completed the survey satisfactorily in wave one and 705 did so in wave two (33 per cent attrition rate). More details about the initial sampling frame and copies of the survey items on political trust can be found in: J. Weinberg, ‘Can political trust help to explain elite policy support and public behaviour in times of crisis? Evidence from the United Kingdom at the height of the 2019–20 coronavirus pandemic’, *Political Studies*, December 2020; https://journals.sagepub.com/doi/10.1177/0032321720980900 (accessed 7 April 2022); data collection was funded by a Leverhulme Trust Early Career Fellowship.

^5^

Legislation.gov.uk, Coronavirus Act 2020, ch.7; http://www.legislation.gov.uk/ukpga/2020/7/enacted (accessed 7 April 2022).

^6^
These scales have strong internal reliability. The Cronbach alpha scores for trust, distrust and mistrust are all well above acceptable thresholds in wave one (0.91, 0.89, 0.81) and wave two (0.92, 0.90, 0.79).

^7^
B. M. Huseby, ‘Government performance and political support. A study of how evaluations of economic performance, social policy and environmental protection influence the popular assessments of the political system’, Department of Sociology and Political Science, Norwegian University of Technology (NTNU), 2000, p. 10.

^8^
See, for example, M. Bisgaard, ‘How getting the facts right can fuel partisan‐motivated reasoning’, *American Journal of Political Science*, vol. 63, 2019, pp. 824–839.

^9^
A marginal effect is the change in the predicted value of a dependent variable (that is, changes in political trust) after changing one independent variable (that is, satisfaction with the government) while all other variables are held at specified values.

^10^
For a review, see J. Citrin and L. Stoker, ‘Political trust in a cynical age’, *Annual Review of Political Science*, vol. 21, 2018, pp. 49–70.

^11^
See, for example, F. Lalot, M. S. Heering, M. Rullo, G. A. Travaglino, D. Abrams, ‘The dangers of distrustful complacency: low concern and low political trust combine to undermine compliance with governmental restrictions in the emerging COVID‐19 pandemic’, *Group Processes & Intergroup Relations*, OnlineFirst, 2021; https://doi.org/10.1177/1368430220967986 (accessed 7 April 2022).

^12^
I use participants’ trust change scores (T1–T2), rather than their trust scores at T2, for three reasons: (1) focussing on relative change rather than isolated scores helps to partial out individual differences in people's use of the response scale; (2) using longitudinal change scores is a more robust test of the causal relationship between the two variables (that is, the results are less likely to be affected by simultaneity or omitted variable bias); and (3) the longitudinal change score is more likely to capture the ‘Covid factor’ (that is, the specific impact of pandemic governance on trust and resultant behaviour).

^13^
Daily and cumulative statistics are available on the Gov.UK Coronavirus Dashboard; https://coronavirus.data.gov.uk/details/vaccinations (accessed 7 April 2022).

^14^
Given that mask wearing is measured using a self‐report scale, the usual caveats about causal inference apply.

^15^
These findings are just one piece of a complex policy puzzle. Omitted variables such as trust in science, threat perceptions, fear of the virus, and personal mental or physical health, are likely to explain as much or more variance in these behaviours as political trust.

